# Pre-elimination stage of malaria in Sri Lanka: assessing the level of hidden parasites in the population

**DOI:** 10.1186/1475-2875-9-25

**Published:** 2010-01-20

**Authors:** Rupika S Rajakaruna, Michael Alifrangis, Priyanie H Amerasinghe, Flemming Konradsen

**Affiliations:** 1 Department of Zoology, University of Peradeniya, Peradeniya, Sri Lanka; 2 Centre for Medical Parasitology at the Department of International Health, Immunology and Microbiology, University of Copenhagen, Copenhagen, Denmark; 3 Department of Infectious Diseases, Copenhagen University Hospital (Rigshospitalet), Denmark; 4 International Water Management Institute, C/o International Crops Research Institute for the Semi-Arid Tropics, Patancheru - 502 324, Hyderabad, Andhra Pradesh, India; 5 Copenhagen School of Global Health, Department of International Health, Immunology and Microbiology, University of Copenhagen, Denmark

## Abstract

**Background:**

With the dramatic drop in the transmission of malaria in Sri Lanka in recent years, the country entered the malaria pre-elimination stage in 2008. Assessing the community prevalence of hidden malaria parasites following several years of extremely low transmission is central to the process of complete elimination. The existence of a parasite reservoir in a population free from clinical manifestations, would influence the strategy for surveillance and control towards complete elimination.

**Methods:**

The prevalence of hidden parasite reservoirs in two historically malaria endemic districts, Anuradhapura and Kurunegala, previously considered as high malaria transmission areas in Sri Lanka, where peaks of transmission follow the rainy seasons was assessed. Blood samples of non-febrile individuals aged five to 55 years were collected from randomly selected areas in the two districts at community level and a questionnaire was used to collect demographic information and movement of the participants. A simple, highly sensitive nested PCR was carried out to detect both *Plasmodium falciparum *and *Plasmodium vivax*, simultaneously.

**Results:**

In total, 3,023 individuals from 101 villages participated from both districts comprising mostly adults between the ages 19-55 years. Out of these, only about 1.4% of them (n = 19) could recall having had malaria during the past five years. Analysis of a subset of samples (n = 1322) from the two districts using PCR showed that none of the participants had hidden parasites.

**Discussion:**

A reservoir of hidden parasites is unlikely to be a major concern or a barrier to the ongoing malaria elimination efforts in Sri Lanka. However, as very low numbers of indigenous cases are still recorded, an island-wide assessment and in particular, continued alertness and follow up action are still needed. The findings of this study indicate that any future assessments should be based on an adaptive sampling approach, involving prompt sampling of all subjects within a specified radius, whenever a malaria case is identified in a given focus.

## Background

Dwindling malaria case counts have placed Sri Lanka in a unique situation to plan towards a pre-elimination programme. Over the past 10 years the number of malaria cases registered at government hospitals have dropped significantly, where cases peaked at 264,549 in 1999 and reduced to 591 in 2006 and then to 196 cases in 2007 [1,2]. The reason for this dramatic decline in malaria cases is not fully understood, but implementation of several large-scale malaria control activities in the island, including increased distribution of insecticide-treated bed nets (ITNs), are likely to have contributed towards the reduction in transmission. However, other factors associated with improved housing, increased access to health services and climatic factors may also have played a role [3].

Sri Lanka has now entered the malaria pre-elimination stage together with ten other countries including Mexico, Iran, Azerbaijan, Georgia, Kyrgyzstan, Tajikistan, Turkey, Uzbekistan, DPR Korea [1]. Elimination is defined as zero incidence of locally acquired malaria infections through deliberate control efforts with continued measures in place to prevent re-establishment of transmission [4]. Prior to implementing an elimination programme, most countries will need to employ some form of active case detection, or a proactive screening of certain segments of the population for malaria parasites [5]. Active detection provides the distinct benefit of enabling treatment of asymptomatic parasite carriers, who are often a major source of continued transmission [5]. A prerequisite in the attempt to eliminate malaria from an endemic area is the identification of parasite carriers that harbour asymptomatic infections that are not noticed, or detection of parasites that have persisted after a certain drug treatment, either because the treatment is not working due to drug resistance or because some stages of the parasites are not targeted by treatment [6]. In an attempt to complement the efforts to eliminate malaria in Sri Lanka, this study assessed the occurrence of malaria parasites in populations free from clinical manifestations during prolonged low malaria transmission hypothesising that populations in districts at high risk, where transmission has been reduced to very low levels in recent years, harbor important reservoirs of asymptomatic parasite carriers.

## Materials and methods

**Figure 1:**
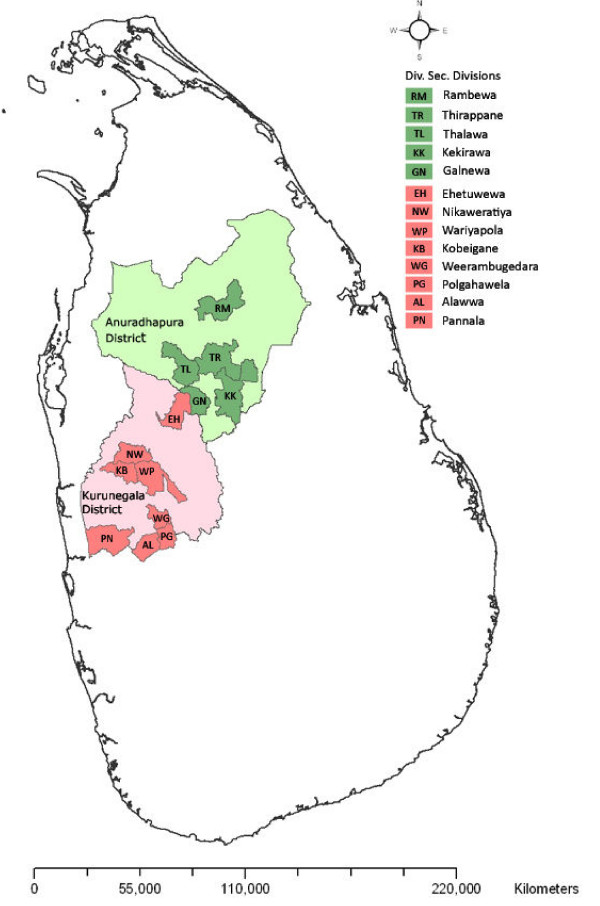
**Map of Sri Lanka showing the selected Divisional Secretary's Divisions (DSDs) in the two districts**.

A cross sectional survey was conducted targeting approximately 3000 individuals living in two malaria endemic districts, Anuradhapura (North Central Province) and Kurunegala (North Western Province) with a history of high malaria transmission (Figure 1). During 2000 to 2006, 70% of the cases found in Sri Lanka have been reported from the districts in war affected areas in the northern and eastern provinces [7]. Among the remaining districts Anuradhapura and Kurunegala reported the highest number of cases for this period having 13,218 and 11,863 cases in 2000, respectively [7]. The high malaria transmission period for both districts is January and July, and the intensity of transmission may vary depending on the rainfall and other factors [8]. Samples were collected from May to August 2007 to include the second peak in July. The government surveillance program reported 12 malaria cases (*Plasmodium falciparum *= 1 and *Plasmodium vivax *= 11) and 14 (*P. falciparum *= 2 and *P. vivax *= 12) from Anuradhapura and Kurunegala for 2007, respectively [9]. Families were selected using the 2001 village census data and computer randomised tables by selecting 25% of the sub-districts (Divisional Secretary's Divisions - DSDs) within the two districts (5/20 DSDs in Anuradhapura and 8/30 DSDs in Kurunegala; Figure 1), and by sampling 15% of the lowest administrative unit (Grama Niladari - GN division), within a sub-district comprising one to three villages (34 GN divisions in 5 DSDs in the Anuradhapura District and 67 GN divisions in eight DSDs in the Kurunegala District; Table 1).

**Table 1:** Details of the samples collected from two districts based on 2001 village census data

District	Divisional Secretary's Divisions (DSD)	Population in DSD	Number of GN Divisions	Number of samples collected	Number of samples analysed by PCR
Anuradhapura	Galnewa	29,989	6	255	117
	Kekirawa	76,003	6	488	217
	Rambewa	31,349	5	226	97
	Thalawa	55,247	7	330	144
	Thirappane	24,508	10	203	84
Kurunegala	Alawwa	64,134	10	216	93
	Ehetuwewa	27,767	5	91	39
	Kobeigane	39,031	5	129	55
	Nikaweratiya	43,110	6	141	57
	Pannala	1,21,895	13	404	176
	Polgahawela	64,030	13	210	99
	Wariyapola	66,572	9	220	97
	Weerambugedara	33,225	6	110	47

Total	13	6,76,860	101	3023	1322

Note: Families were selected using computer randomised tables by selecting 25% of the Divisional Secretary's Divisions (DSDs) within the two districts and by sampling 15% of the Grama Niladari (GN) divisions within a DSD.

In each GN division, houses were visited during daytime by a team from the Anti-malaria Campaign, supervised by the Regional Malaria Officer of each district. All residing individuals between the ages of five to 55 years were sampled after informed consent, if they agreed to participate. Parents/guardians were asked on behalf of children. Age group was selected based on the high risk groups and likely to have hidden parasites because of tolerance. Houses on both sides of the road in a village were visited until a representative number from each village was covered and the target number of samples was reached. Demographic and household information and malaria history of the participants were collected by means of a questionnaire. Information on disease history, episodes of malaria for the past five years, treatment sought and movement related to occupation during the past six months was included in the survey form. Four drops of blood were spotted onto filter paper (Whatman #3) from a finger prick and were kept at -20°C in separately sealed plastic bags with silica gel until use.

The study protocols for collection of blood samples and information were approved by the Ethical Committee of the Faculty of Medicine, University of Peradeniya, Sri Lanka.

### Identification of *Plasmodium falciparum and Plasmodium vivax *infections by nested PCR

Genomic DNA was extracted from the blood samples using a chelex-100 method. A simple nested PCR was developed to diagnose both *P. falciparum *and *P. vivax *infections in samples by amplification of the gene coding for the small subunit of ribosomal RNA based on the original protocol [10]. The outer primers used were rPLU5 and rPLU6 [10], while the primers used in the nested PCR were rPLU-N1-FW: 5'-GAATACTACAGCATGGAATAACAA and rPLU-N2-RV: 5'-CAAAGACTTTGATTTCTCATAAG. The nested primers amplified sequences of 369 bp and 352 bp for *P. falciparum *and *P. vivax *infections, respectively that could be easily separated by agarose gel electrophoresis. The 10 μL PCR master mix consisted of 0.063 μM primers (outer PCR) or 0.25 μM primers (nested PCR), 5 μL Tempase master mix [TEMPase Hot Start Master Mix (Ampliqon III, VWR-Bie Berntsen, Denmark)], 1 μL of extracted DNA (or 1 μL of outer PCR product) and H_2_O. The PCR conditions for the outer PCR were: Initial activation of Tempase enzyme at 94°C for 15 min, followed by 30 cycles of 94°C for 1 min, 58°C for 2 min and 65°C for 2 min and finally 58°C for 2 min and 65°C for 5 min. For the nested PCR: 94°C for 15 min, followed by 30 cycles of 94°C for 30 sec, 57°C for 1 min and 65°C for 1 min and finally 58°C for 2 min and 65°C for 5 min. All PCR reactions were run in Eppendorf 96-well PCR plates with foil sealing (AH Diagnostics, Denmark) minimizing contamination risks and evaporation. Positive *P. falciparum *and *P. vivax *isolates as well as negative controls, extracted DNA from persons living in Denmark with no history of malaria were included in all PCR reactions, both to ensure a successful PCR and to check for contamination. All samples were run on 1.5% ethidium bromide-containing agarose gels and visualised under UV-light. In case of doubt, for instance if mixed species infections were suspected, the original protocol for species identification using *P. falciparum *or *P. vivax *specific primers and PCR conditions were used as described in reference [10].

The sensitivity of the nested PCR was evaluated by measuring the threshold of sensitivity on a dilution series of *P. falciparum *laboratory isolate FCR3. The FCR3 isolate was grown to 5,800 parasites/μl and diluted in full blood to 1000, 100, 10, 5, 2.5, 1.25, 0.6, 0.3 and 0.15 parasites/μl. This dilution series was dotted onto Whatman filter paper #3 and the blood spots were extracted similarly to the samples by use of the chelex-100 method.

Subsets of samples were selected using computer generated randomised tables and analysed. First, 25% was selected from the initial sample and then another 25% from the remaining sample was analysed. Since all 1,322 samples tested turned out negative rest of the sample was not analysed.

## Results

In total, 3,023 participants from 101 villages constituting of mostly adults between the ages 19-55 years (73%, Table 2) were enrolled in the study from both districts. Of these, only about 1.4% of them (n = 19) reported to have had microscopically or RDT confirmed malaria during the past five years (Table 2). The low number of malaria episodes recorded during the past five years is a reflection of the extremely low levels of transmission during this period. It is noted that responses could also be influenced by respondent recall, the way the questions were posed by the interviewer or perceived by the respondent.

**Table 2:** Demographic information and malaria history of the participants from two districts

		District		
			
		Kurunegala % (n = 1521)	Anuradhapura % (n = 1502)	Total %
Sex	Male	43	44	43
	Female	57	56	57
Age	5-12 years	7	16	12
	13-18 years	13	13	13
	19-55 years	80	71	76
Malaria	History of malaria	21	27	24
	Malaria during last 5 years	5	6	6
Travel to malarious areas	During last month	14	34	24
	During last six months	90	60	75

The molecular diagnostic analysis of a subset (1,322) of blood samples revealed that none of the samples were positive for *P. falciparum *or *P. vivax*. This corroborates the current observations that the transmission is extremely low.

## Discussion

The results show that there are no hidden parasites in the population analysed. According to the AMC (Sri Lanka), for 2007 only 12 positive cases (microscopically confirmed) were reported from the Anuradhapura District (total population 791,000) while 14 cases were reported from the Kurunegala District (total population 1,511,000). Of the 12 cases in the Anuradhapura District, 11 cases were indigenous while the remainder had contracted the disease during visits to other malarious districts within Sri Lanka, specifically to Trincomalee (AMC, Sri Lanka, unpublished data). Only a small percentage (1.4%) of the population tested had microscopically or RDT confirmed malaria during the past five years. In Sri Lanka, microscopically confirmed blood smears or rapid diagnostic kits are standard diagnostic procedures adopted before the prescription of malaria drugs to patients. Since the medical services at government hospitals are supported by qualified staff, in general, medication is initiated only after a blood test and most patients appear to prefer this approach [11]. According to Cosmetic Devices and Drug Act (1979) private or government drug outlets are not permitted by law to issue any anti-malarial drugs without a prescription or a confirmed blood slide. All the respondents who had malaria claimed that they had been tested and confirmed before treatment.

At a pre-elimination phase, it is important to determine the occurrence of hidden parasite reservoirs by using sensitive and robust diagnostic tools. The nested PCR method used in this study was a simple, relatively cheap and sensitive method capable of identifying *P. falciparum *and *P. vivax *parasite infections simultaneously in blood samples. The sensitivity of the nested PCR was assessed by determining the threshold of positivity on a dilution series of *P. falciparum *laboratory isolate, FCR3. The method could detect 1.25 parasites/μl, which is roughly 30 parasites in a 25 μl blood spot. When using the original primers for detecting *P. falciparum *only, as described in reference [10] similar sensitivity was found. Given this sensitivity, very low levels of parasitaemia would not have gone undetected. Other simple PCR methods have as well been described with similar sensitivity. For instance, recently a novel technique known as Loop-mediated isothermal amplification (LAMP) that rapidly amplifies target DNA under isothermal conditions has been described with similar sensitivity for the detection of *P. vivax *infections as when using nested PCR [12]. However the detection limit for nested PCR was lower (30 ± 5 parasites/μl vs. 3 ± 5 parasites/μl for the LAMP and nested PCR, respectively) [12] and this difference may become important when detecting the occurrence of sub-microscopical infections.

The method used here was a sensitive diagnostic tool to test the blood stages of the parasite, and to the best of our knowledge there are no tests to detect the liver stages of the parasite. Since *P. vivax *can form undetected hypnozoites, there is a risk that such a reservoir persists in the indigenous population. Nevertheless, the contribution of these dormant stages in causing outbreaks is limited in Sri Lanka under the present policy of co-administering primaquine in combined with the first line anti-malarial drug in the treatment of uncomplicated cases.

The samples collected for this study used a random method in selecting the study areas with the aim of assessing community prevalence. This sampling method picked out 60% (3/5) and 50% (4/8) of high prevalence DSDs from Anuradhapura and Kurunegala districts, respectively (based on malaria data from 1995 to 2005; AMC personal communication). However, with the lack of parasites identified from this approach and the overall very low community reported history of malaria, the future design of assessments to identify hidden parasites could be changed to focus on villages where a case of malaria had been reported within e.g. the past three months and in a broader age group. This approach would of course not address the objective set for this study but would still provide public health relevant information. Whether a different method of sampling where selection of geographical areas with a history of high transmission would have given different results, remains to be investigated. However, it is doubtful, if such a study were to change the current conclusion drastically, given such low case counts even in the high transmission areas. During the study period only one indigenous malaria case (*P. vivax*) from Anuradhapura district was diagnosed (microscopically) by AMC and treated. Unfortunately, the sample was lost for verification by our PCR method. However, 12 blood samples that were collected from the neighbourhood were all negative. The approaches to active case detection involve screening family members and neighbours of new cases, screening targeted individuals near (perhaps within 1 km) new cases, periodic perhaps monthly screening of targeted communities where residual transmission and continuing cases are reported [5]. According to Malaria Elimination Group (MEG) Prospectus on Elimination, there is currently no evidence to suggest that the approaches on the right end of the spectrum (i.e., mass screening) are more effective and/or cost-effective than the more-limited measures [5]. The MEG therefore recommends that countries only adopt these measures following detailed analysis of feasibility and cost-effectiveness [5].

Aiming at elimination of malaria further implies the need for effective surveillance strategies to monitor progress. In the case of Sri Lanka, historical evidence shows that malaria incidence can fluctuate markedly. Thus, a well focused system of surveillance that will identify emerging malaria outbreaks and tools in place for prompt treatment is required although the case rates are dwindling. Further, the current knowledge on parasite resistance to anti-malarials [13] in the country has contributed to the selection of the most suitable and sensitive drugs. Artemisinin combination therapy (ACT) is now the first-line drug for uncomplicated falciparum malaria in most of the malaria endemic world. Artemisinins kill nearly all of the asexual stages of parasite development in the blood, and also affect the sexual stages of *P. falciparum *(gametocytes), which transmits the infection to mosquitoes, but do not affect pre-erythrocytic development. Hence, a critical part of malaria elimination is assessing the prevalence of hidden parasites. However, even extremely low levels of circulating parasites in the population or the introduction of parasites by individuals migrating into Sri Lanka may still generate an outbreak with the potential for large scale epidemics if the vector population reaches very high levels. Therefore, vector surveillance, especially following the reduced efforts of indoor residual spraying, is an important aspect to be dealt with side by side with the detection of hidden parasites. This study documents an approach that can be extended to a national scale that will enable the collection of comprehensive data sets for future planning and policy formulation.

Based on this analysis, the reservoirs of hidden parasites may not be a major concern or barrier to elimination efforts, but calls for an island-wide assessment and continued alertness, in particular, in areas that were affected by the civil war. Additionally, using an adaptive sampling approach for prompt screening of family members and neighbors of new cases can be carried out to confirm any malaria hotspots [14] before in-depth elimination strategies are implemented.

## Competing interests

The authors declare that they have no competing interests.

## Authors' contributions

RSR acquired and analysed data, carried out the lab work and wrote the manuscript. MA contributed to study design and interpretation of data, supervised DNA work, revised the manuscript for important intellectual content. PHA and FK contributed to concept development and study design, edited and revised the manuscript critically for content. All authors read and approved the final manuscript.
